# Abnormally Large Synovial Herniation Resulting in Progressive Rocker Bottom Deformity in a Patient with Idiopathic Midfoot Charcot Neuroarthropathy: A Case Report

**DOI:** 10.7759/cureus.6267

**Published:** 2019-12-01

**Authors:** Craig Verdin, Nilin M Rao

**Affiliations:** 1 Department of Orthopedic Surgery, Podiatry Division, Cleveland Clinic Foundation, Cleveland, USA; 2 Podiatric Medicine and Surgery, Highlands-Presbyterian/St. Luke’s Medical Center, Denver, USA

**Keywords:** charcot neuroarthropathy, synovial cyst, rocker-bottom deformity, idiopathic, neuropathy

## Abstract

Deformities associated with Charcot neuroarthropathy (CN), a rare and devastating post-diabetic complication, place patients at risk for infection and amputation. In the present case, a 56-year-old male with idiopathic CN presented with complaints of progressively growing plantar soft tissue deformity. Routine hematology, biochemistry, paracentesis, and fluid analysis were conducted. The patient was treated conservatively and will continue to be monitored for progression of the condition.

## Introduction

Charcot neuroarthropathy (CN) of the foot and ankle, also known as Charcot joint, Charcot neuropathic osteoarthropathy, or Charcot foot, is a serious and limb-threatening complication that typically affects 1% of patients with longstanding neuropathy of differing etiology, and places patients at risk for adverse outcomes [1-3]. The most common causes of CN include diabetes mellitus, syphilis, rheumatoid arthritis, chronic alcoholism, congenital causes of neuropathy, familial amyloid polyneuropathy, and even iatrogenic causes [4,5]. The rare condition, first described by Jean-Martin Charcot, a French neurologist, is a complex condition that continues to be a challenge to manage both surgically and conservatively. While this condition can occur anywhere in the body, it is commonly found in the foot and ankle likely due to a combination of contributing factors such as weight bearing on an insensate limb, ligamentous laxity, and, theoretically, the presence of a well-perfused hypervascular environment [6,7]. The condition is characterized by osseous destruction of bone and joint architecture that leads to eventual joint subluxation and pathologic fractures, which can result in ulceration, infection, and amputation. CN is often described as an inflammatory condition resulting from unperceived repetitive microtrauma [8]. Clinically, patients with CN typically present with non-specific changes often described as a “red, hot, and swollen” foot and potentially a rocker-bottom structural deformity [9-11]. We report a unique case of a patient with idiopathic midfoot CN, who is presented with complaints of an increasing rocker bottom deformity and discomfort due to a communicating synovial cyst.

## Case presentation

**Figure 1:**
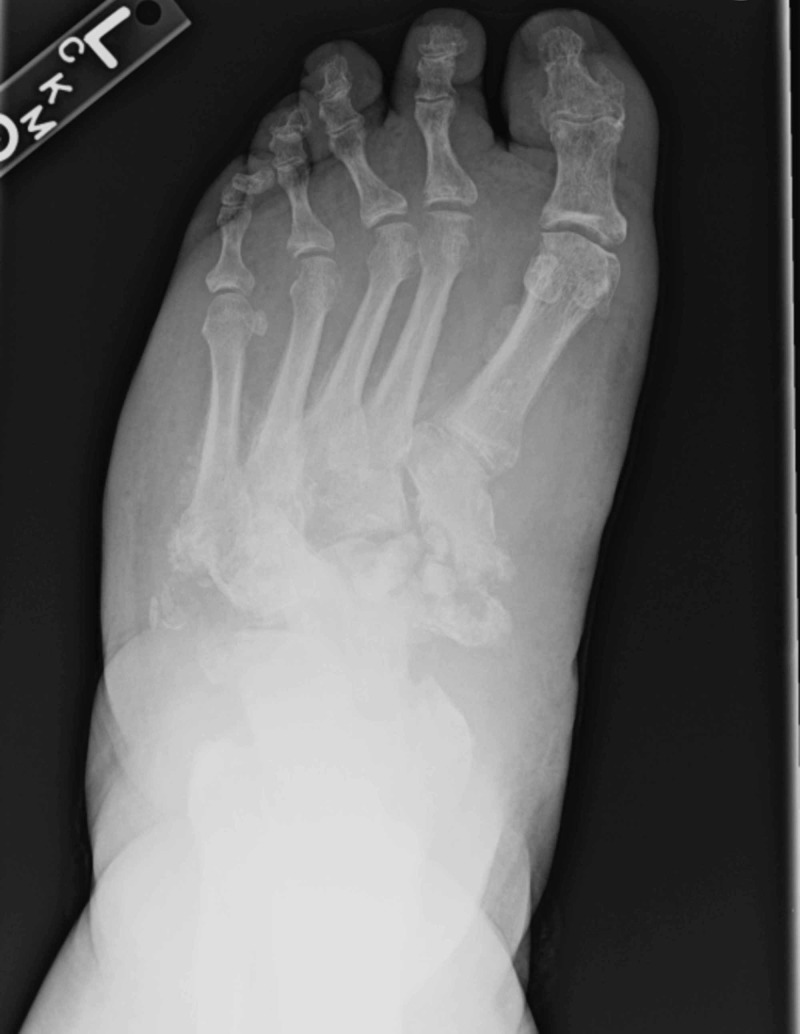
Anteriorposterior foot view demonstrated diffuse tarsal bone fragmentation and obliteration of native joint spaces consistent with active Charcot neuroarthropathy.

**Figure 2:**
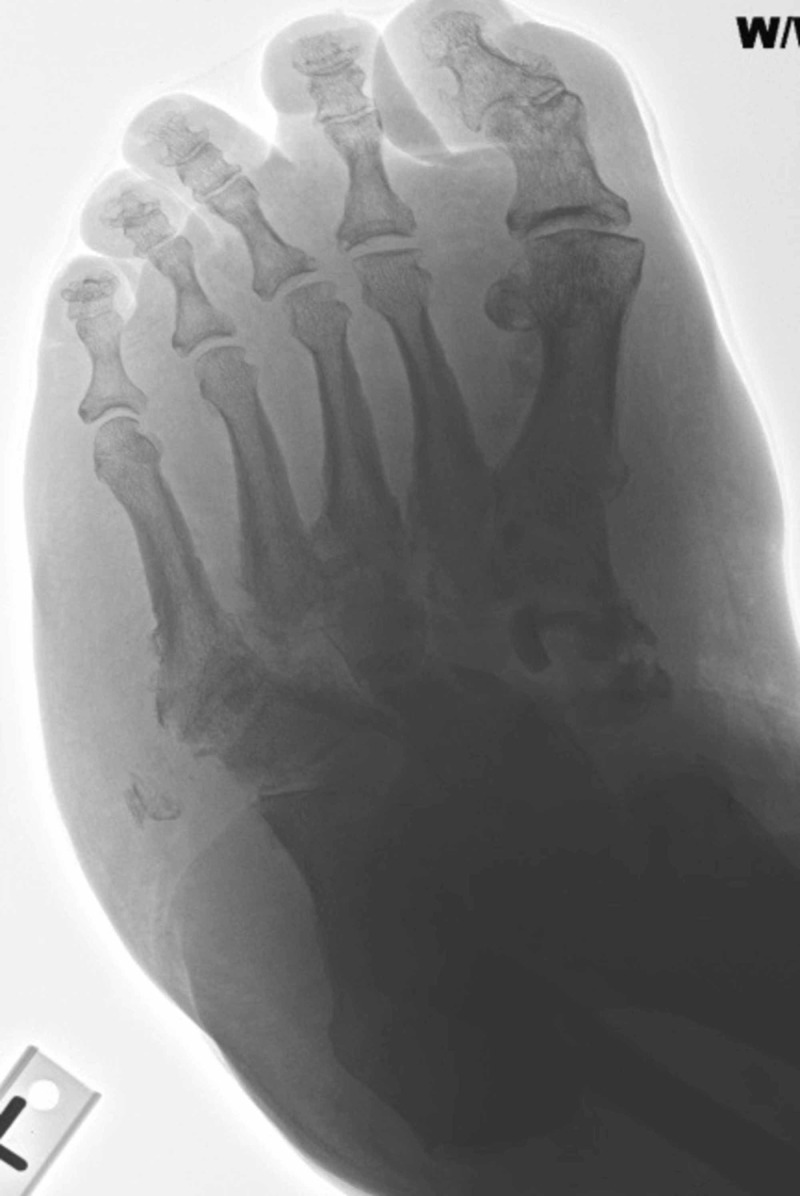
Medial oblique view demonstrated diffuse tarsal bone fragmentation and obliteration of native joint spaces consistent with active Charcot neuroarthropathy.

**Figure 3:**
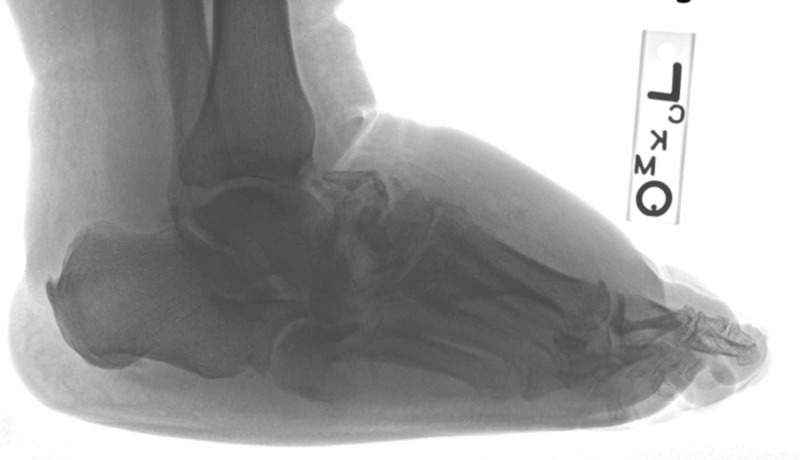
Lateral view demonstrated lateral foot view demonstrated comminuted midfoot fracture pattern, disorganization, and swelling consistent with active Charcot neuroarthropathy.

A 56-year-old non-diabetic male with CN presented with symptoms of new-onset pain and concerns about progressive volumetric changes of the plantar rocker bottom deformity of his left midfoot over a six-month period. He had a past medical history significant for hyperlipidemia and tobacco use and was previously diagnosed with CN, resulting in midfoot collapse, after a traumatic forefoot injury that occurred in June 2016 (Figures 1-3).

**Figure 4:**
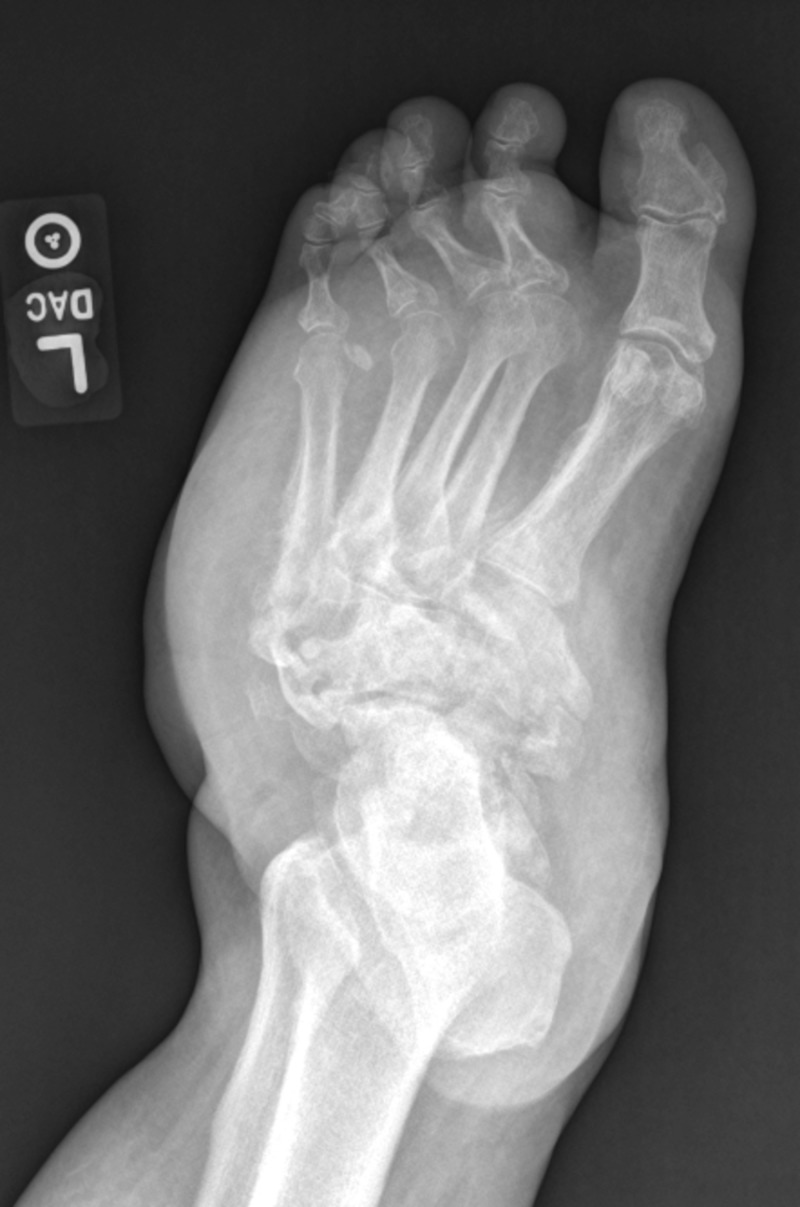
Anteriorposterior view demonstrated consolidation of bone fragments and presence of joint arthrosis indicating an inactive process.

**Figure 5:**
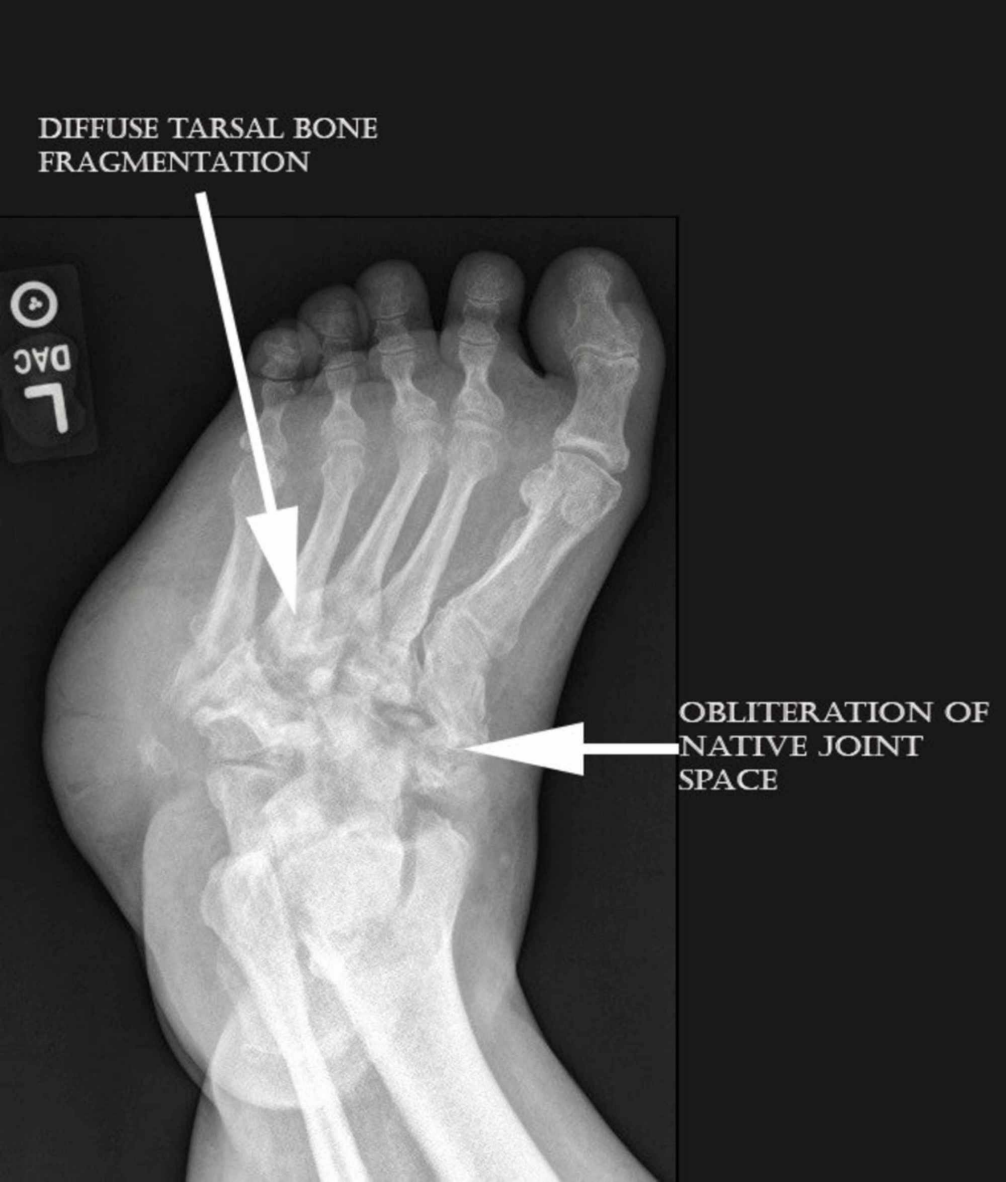
Medial oblique foot view demonstrated consolidation of bone fragments and presence of joint arthrosis indicating an inactive process. Also of note is the radiodense plantar lateral mass at the level of the midfoot.

**Figure 6:**
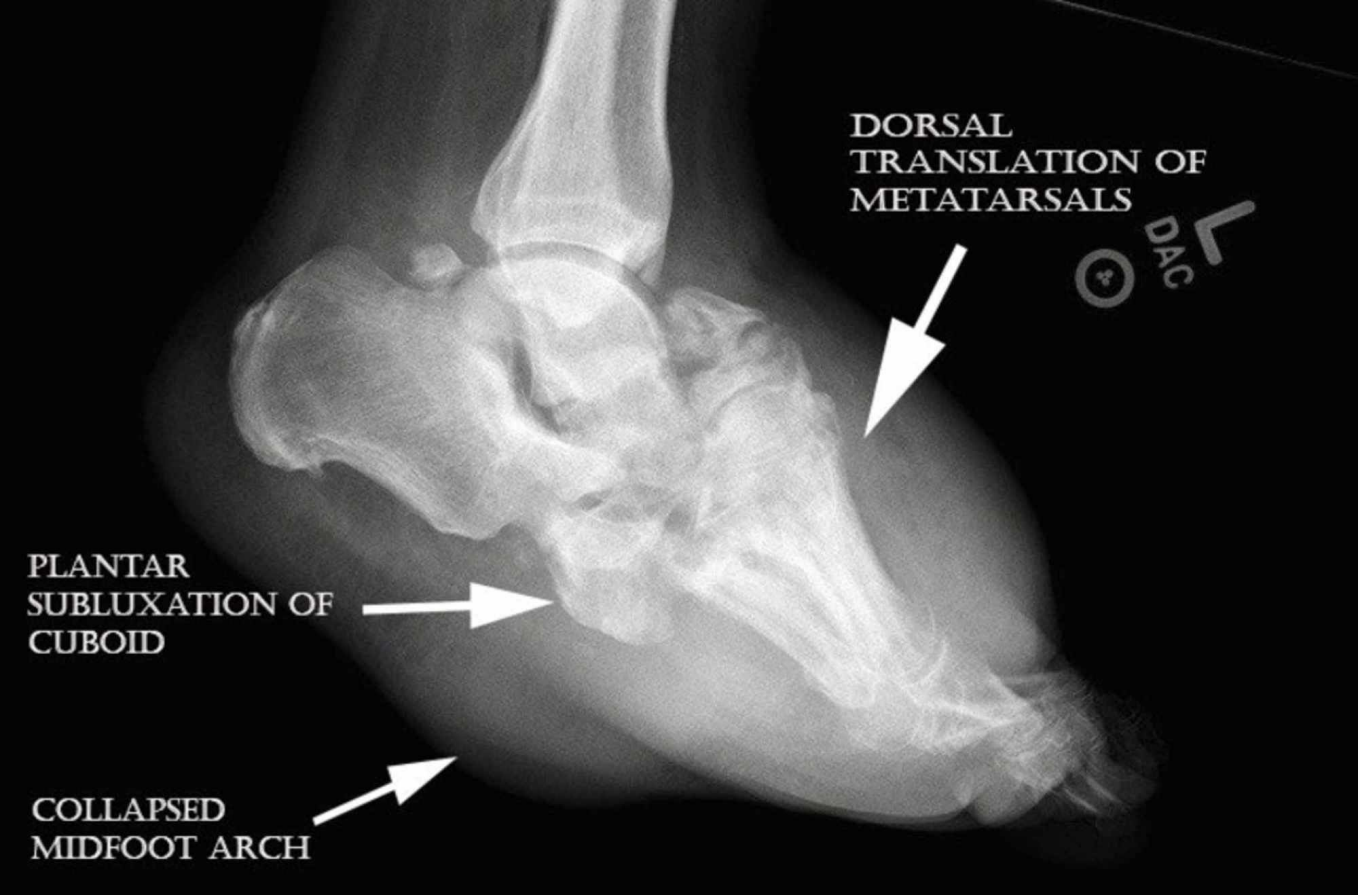
Lateral left foot view showed dorsal translation of metatarsals, mild cuboid subluxation, and rocker bottom deformity.

He had been treated conservatively with the use of an offloading CROW boot, but reported increasing discomfort when placing his foot in the offloading device, believing it does not fit him anymore. The enlargement was most prominent on the plantar lateral aspect of the midfoot. The patient did not report any recent trauma, increased weight bearing, or plantar ulcerations. On physical examination, the left foot showed a 14 cm long and 8 cm thick, fluctuant, non-tender, non-illuminating, non-mobile, gel-like mass over the plantar lateral left midfoot. No palpable bony prominences or open lesions in the skin were noted. The left foot did not show any signs of increased warmth relative to the contralateral limb, erythema, calor, crepitus, or signs of infection. The patient’s dorsalis pedis pulse was palpable, the posterior tibial pulse was non-palpable, and loss of protective sensation to a 5.07 g Semmes-Weinstein monofilament was noted. Radiographs of the left foot were obtained and revealed no new osseous changes or progression of deformity; however, mild cuboid plantar subluxation was noted. These findings are consistent with inactive and stable CN; however, a substantial increase in soft tissue density with an associated plantar lateral compartment expansion was noted, suggesting fluid collection (Figures 4-6).

**Figure 7:**
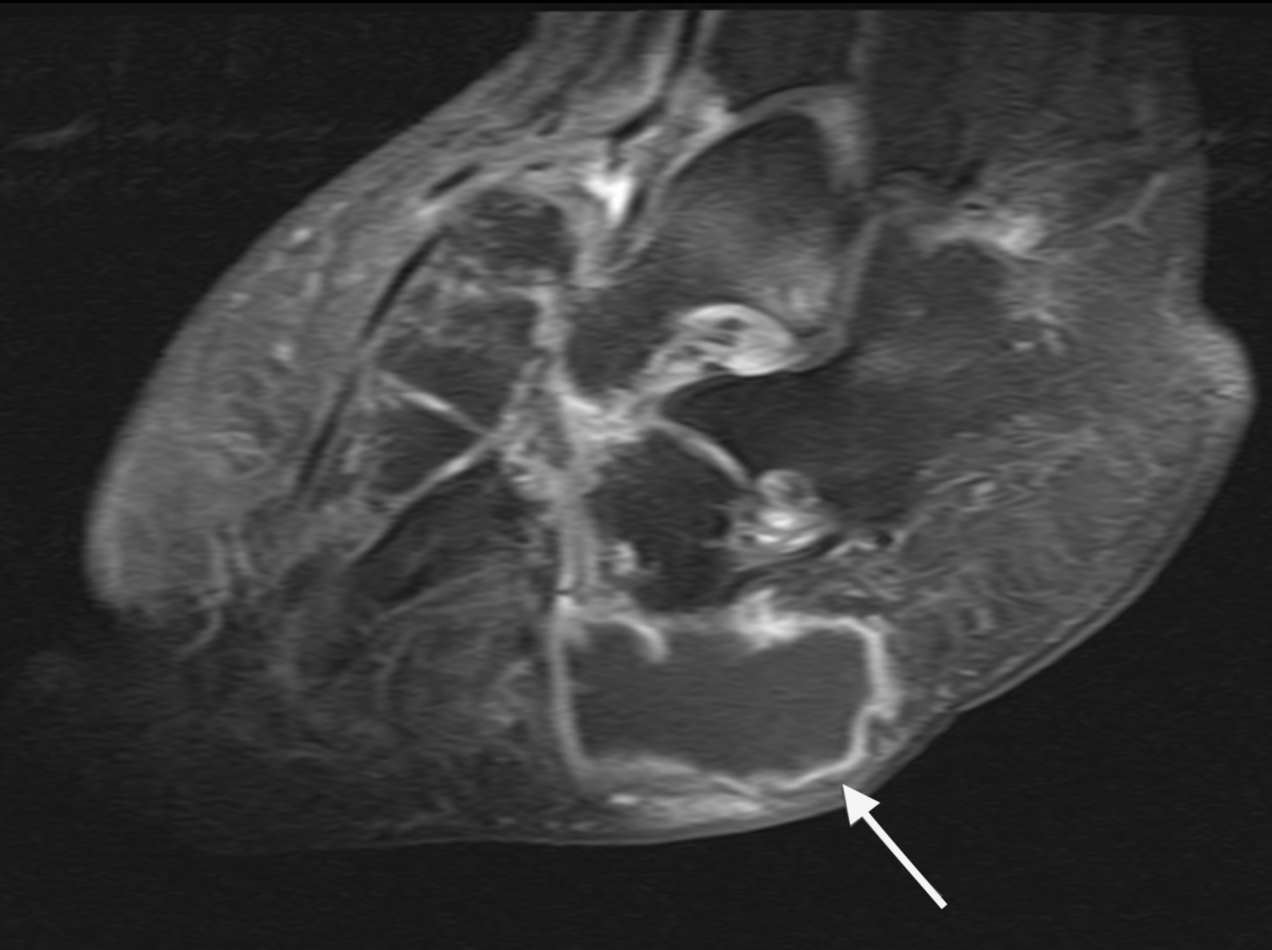
Sagittal MRI illustrated a well-organized large heterogeneous thick rim-enhancing fluid collection within the plantar lateral tissue.

Advanced imaging was ordered, and an MRI of the afflicted limb further confirmed a well-organized large heterogeneous thick rim-enhancing fluid collection within the plantar lateral tissue (Figure 7).

**Figure 8:**
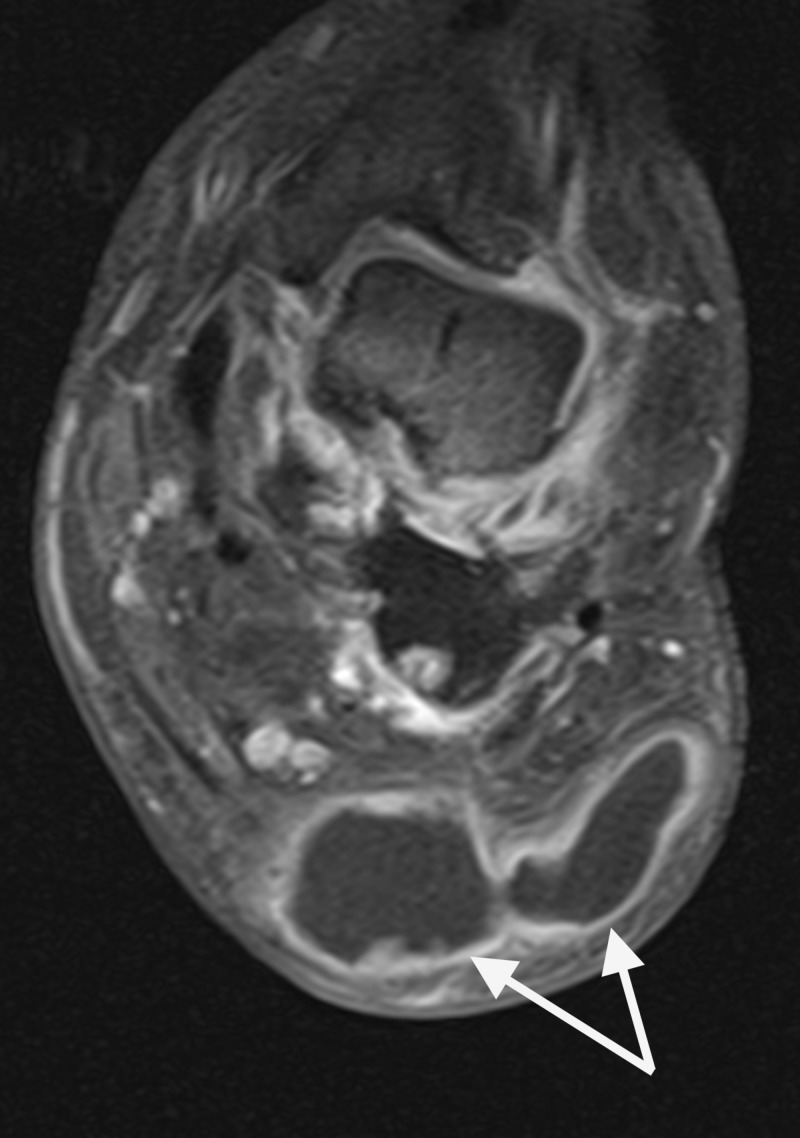
Coronal MRI illustrated a well-organized large heterogeneous thick rim-enhancing fluid collection within the plantar lateral tissue.

**Figure 9:**
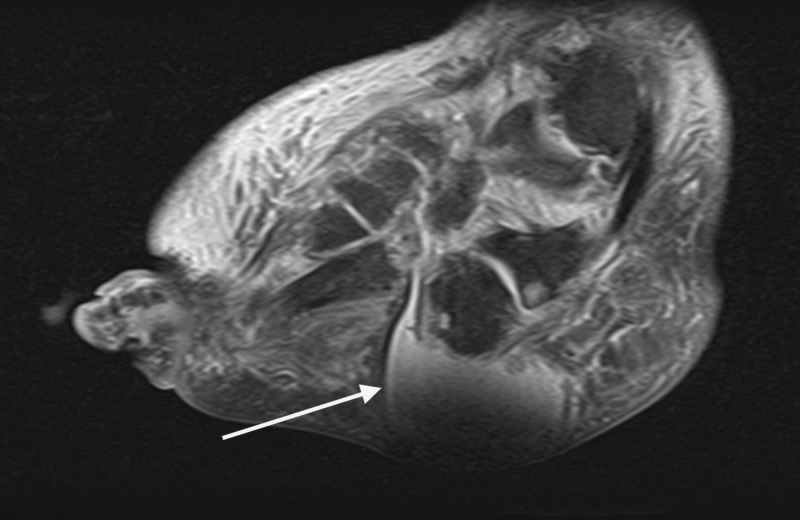
Sagittal MRI revealed well-organized fluid filled lesion within the plantar lateral tissue that is communicating with the disrupted joints of the midfoot.

**Figure 10:**
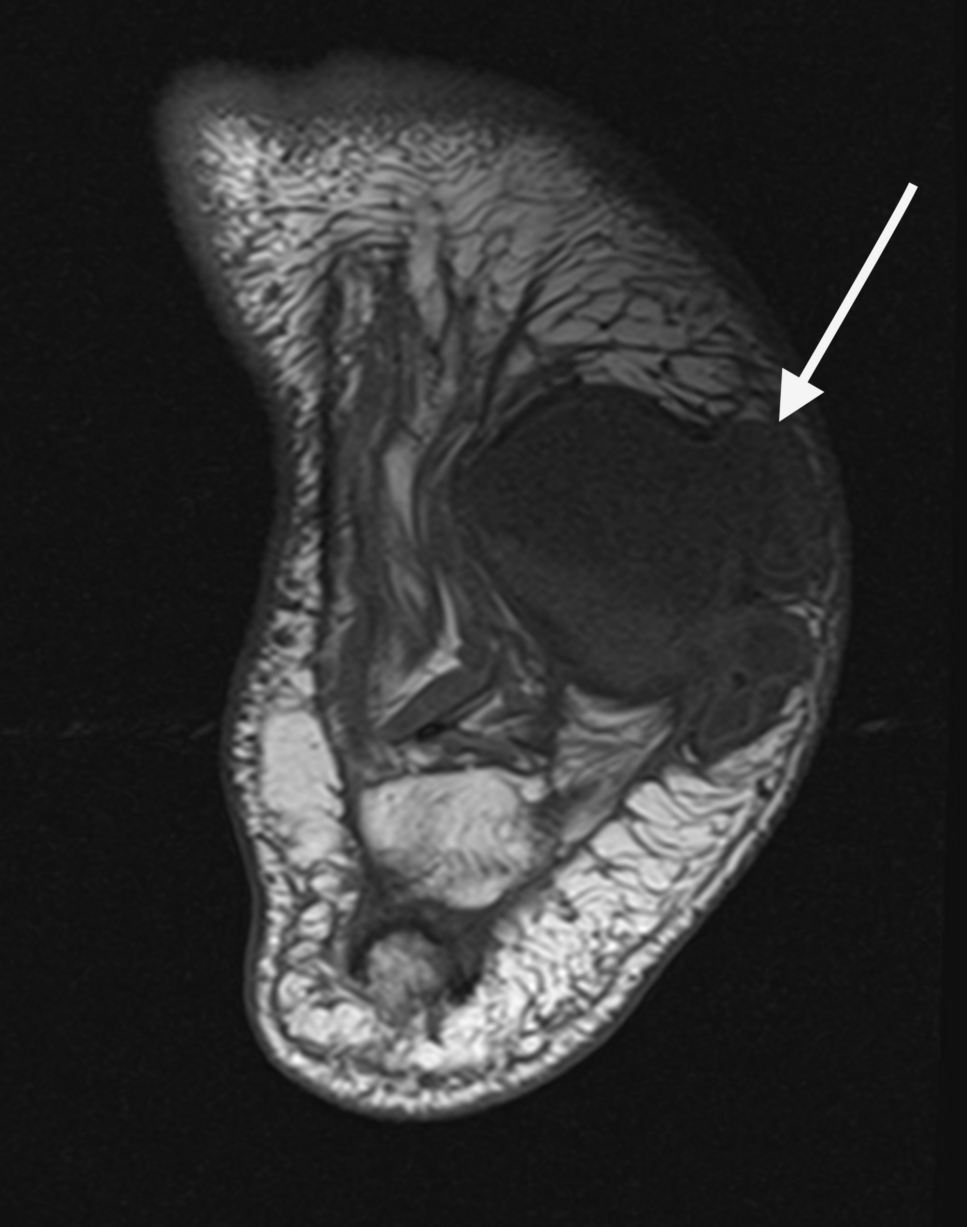
Axial MRI revealed well-organized fluid filled lesion within the plantar lateral tissue that is communicating with the disrupted joints of the midfoot.

The fluid collection was plantar to the cuboid and calcaneus, communicating with the midfoot joint capsules, and measuring 8 cm in the x-direction and 4 cm in the y-direction (Figures 8-10).

Despite the lack of a wound or source of infection, infection was considered in the differential diagnosis but was considered highly unlikely due to patient’s relatively unremarkable inflammatory markers (white blood cell [WBC]=8.38 K/cmm, erythrocyte sedimentation rate=26 mm/hr, procalcitonin<0.05 ng/mL, lactic acid=2.0 mmol/L). After consent was obtained, paracentesis of the plantar lateral midfoot was performed to rule out potential malignancy and further confirm lack of infection. Paracentesis of the plantar mass yielded 30 mL of a yellow hazy serous fluid, with a WBC count of 185 white blood cells/mm^3^ (5% lymphocytes, 1% polymorphonuclear cells, 43% monocytes), and no malignant cells or crystals present. In addition, direct stains and cultures were performed and were negative. In conclusion, the diagnosis of the lesion was a benign synovial cyst communicating with the disrupted capsules of the midfoot. While surgery was contemplated to encourage a functional outcome, it was concluded that risks outweighed the benefits and the patient was at risk for delayed wound healing due to the presence of persistent synovial fluid drainage, resulting in a progressive rocker bottom deformity [12]. Since the vast majority of patients with CN have been successfully treated conservatively through protected offloading, the decision was made to recast the patient for a new CROW walker for sustained offloading [13]. Continued monitoring for the progression of the deformity and development of an ulceration or infection would become a priority through frequent follow-up appointments and use of other interventions, if needed [14]. On his three-month follow-up after diagnosis, and after receiving a new CROW to accommodate the volumetric changes, the patient’s soft tissue deformity was stable, the patient does not admit to any new changes to his foot or overall condition, and no issues were noted at the 2.5-year mark.

## Discussion

Synovial cysts are benign, gelatinous fluid accumulations that are usually found in subcutaneous tissue and are derived from disrupted joint capsules or tendon sheaths, allowing for the accumulation of synovial fluid outside of the capsule. They are most commonly found around joints in wrist, elbows, shoulders, knees, and the foot [15]. While more common elsewhere in the body, Kirby *et al.* demonstrated that 32% and 48% of synovial cysts in the lower extremity were found in the ankle and dorsum of the foot, respectively [16]. In the present case, we present a case in which a massive synovial cyst is found to be communicating with the joints of the midfoot, resulting in a plantar displacement of synovial fluid. To our knowledge, no other reports of an abnormally large synovial cyst secondary to midfoot collapse, resulting in a progressive soft tissue rocker bottom deformity, have been reported in the literature. Synovial cysts are common in the setting of other inflammatory arthropathies, especially rheumatoid arthritis [17]. The etiology of how synovial cysts occur is not well understood, however, trauma in or outside of joints in addition to subsequent inflammation is believed to be a factor in the pathogenesis of such formations. It is the belief of the authors that this case report is novel because it describes a unique presentation of a characteristic and progressing rocker bottom deformity, in a patient with inactive CN, that cannot be attributed to osseous changes and presence of bony prominence. Without subluxation of the stable midfoot, it is unusual for patients to present with a worsening deformity. A possible explanation for this occurrence is that there was an overproduction of synovial fluid during the active inflammatory stage of Charcot. This may have caused the midfoot joint capsules to further become weak or distended, and in the setting of osseous architectural and degenerative joint destruction in an insensate weight-bearing limb, this progression likely allowed for plantar communication and displacement of synovial fluid. While not the case in this case report, the further distention of the plantar deformity due to draining synovial fluid places the diabetic patient at risk for ulceration, subsequent infection, and potential amputation. Because the presence of synovial cysts typically do not present any risk to patients, treatment of synovial cysts is largely palliative in the setting of discomfort or pain. Aspiration of synovial accumulations in an effort to decompress often results in recurrence of cysts due to failure to address source of synovial displacement, therefore, surgical excision of the cyst in addition to removal of degenerative capsules is essential to successfully treating this pathology [18,19].

## Conclusions

Regardless of the causative nature of the rocker bottom deformity, whether it be cystic or osseous in nature, conservative and surgical management of CN is an increasingly challenging task that requires advanced knowledge of the condition itself, presence of complicating comorbidities, and surgical skill.
